# Biodistribution and post-therapy dosimetric analysis of [^177^Lu]Lu-DOTA^ZOL^ in patients with osteoblastic metastases: first results

**DOI:** 10.1186/s13550-019-0566-x

**Published:** 2019-11-28

**Authors:** Ambreen Khawar, Elisabeth Eppard, Frank Roesch, Hojjat Ahmadzadehfar, Stefan Kürpig, Michael Meisenheimer, Florian. C. Gaertner, Markus Essler, Ralph. A. Bundschuh

**Affiliations:** 1 Department of Nuclear Medicine, University Medical Center Bonn, Bonn, Germany; 2 Institute of Nuclear Chemistry, Johannes Gutenberg-University Mainz, Mainz, Germany

**Keywords:** [^177^Lu]Lu-DOTA^ZOL^, Organ absorbed doses, Bone seeking therapeutic radionuclides, Prostate carcinoma, Bronchial carcinoma

## Abstract

**Background:**

Preclinical biodistribution and dosimetric analysis of [^177^Lu]Lu-DOTA^ZOL^ suggest the bisphosphonate zoledronate as a promising new radiopharmaceutical for therapy of bone metastases. We evaluated biodistribution and normal organ absorbed doses resulting from therapeutic doses of [^177^Lu]Lu-DOTA^ZOL^ in patients with metastatic skeletal disease.

**Method:**

Four patients with metastatic skeletal disease (age range, 64–83 years) secondary to metastatic castration-resistant prostate carcinoma or bronchial carcinoma were treated with a mean dose of 5968 ± 64 MBq (161.3 mCi) of [^177^Lu]Lu-DOTA^ZOL^. Biodistribution was assessed with serial planar whole body scintigraphy at 20 min and 3, 24, and 167 h post injection (p.i.) and blood samples at 20 min and 3, 8, 24, and 167 h p.i. Percent of injected activity in the blood, kidneys, urinary bladder, skeleton, and whole body was determined. Bone marrow self-dose was determined by an indirect blood-based method. Urinary bladder wall residence time was calculated using Cloutier’s dynamic urinary bladder model with a 4-h voiding interval. OLINDA/EXM version 2.0 (Hermes Medical Solutions, Stockholm, Sweden) software was used to determine residence times in source organs by applying biexponential curve fitting and to calculate organ absorbed dose.

**Results:**

Qualitative biodistribution analysis revealed early and high uptake of [^177^Lu]Lu-DOTA^ZOL^ in the kidneys with fast clearance showing minimal activity by 24 h p.i. Activity in the skeleton increased gradually over time. Mean residence times were found to be highest in the skeleton followed by the kidneys. Highest mean organ absorbed dose was 3.33 mSv/MBq for osteogenic cells followed by kidneys (0.490 mSv/MBq), red marrow (0.461 mSv/MBq), and urinary bladder wall (0.322 mSv/MBq). The biodistribution and normal organ absorbed doses of [^177^Lu]Lu-DOTA^ZOL^ are consistent with preclinical data.

**Conclusion:**

[^177^Lu]Lu-DOTA^ZOL^ shows maximum absorbed doses in bone and low kidney doses, making it a promising agent for radionuclide therapy of bone metastasis. Further studies are warranted to evaluate the efficacy and safety of radionuclide therapy with [^177^Lu]Lu-DOTA^ZOL^ in the clinical setting.

## Introduction

Occurrence of painful bone metastases is a frequent complication of solid tumors that reduces the quality of life in many patients [1]. Radionuclide therapy for bone pain palliation in patients with progressive skeletal metastatic disease has been established in the clinical routine already decades ago [2, 3]. Several radiopharmaceuticals are or have been in use such as [^32^P]Na_2_P, [^89^Sr]SrCl_2_, [^153^Sm]Sm-EDTMP, and [^186^Re]Re-HEDP, [^117m^Sn]SnCl_2_, and [^223^Ra]RaCl_2_ [1, 2, 4]. The therapeutic effect requires the accumulation of these radiopharmaceuticals at osteoblastic sites of metastases and deposition of energy by β^−^ or α particle. These radiopharmaceuticals are used either as monotherapy or in combination with systemic chemotherapy and bisphosphonates [4]. The choice of the radiopharmaceutical is dependent on its inherent properties such as physical half-life, energy and particle range, as well as ease of availability, efficacy, and side effects [5].

Yet, the quest for a stable and effective bone-seeking therapeutic radiopharmaceutical is still an ongoing process. Lutetium-177 with a half-life of 6.73 days, a low range of its β^−^ particles with maximum energy (*E*_βmax_ = 497 keV), gamma emissions at energies of 112 keV (6.4%) and 208 keV (11%), and the possibility of cost-effective large scale production with high specific activity and radionuclide purity has gained high acceptance as a therapeutic radionuclide [6]. Owing to the deposition of its β^−^ energy in the lesions and their close environment, it is best suited for small- to medium-sized tumor lesions when labeled with a suitable carrier [7]. [^177^Lu]Lu-DOTA-TOC, [^177^Lu]Lu-DOTA-TATE [8, 9], and [^177^Lu]Lu-PSMA-617 [10, 11] have been proven effective for the treatment of neuroendocrine tumors and metastatic castration-resistant prostate carcinoma (mCRPC), respectively. Moreover, they allow for a good theranostic combination with their gallium-68-labeled imaging counterparts using positron emission tomography (PET) [12].

Bisphosphonates with antiresorptive properties [13] have also been labeled with lutetium-177. Among these are [^177^Lu]Lu-DOTMP [14, 15], [^177^Lu]Lu-EDTMP [16–21], and [^177^Lu]Lu-BPAMD [22–25]. Phase I and II studies with [^177^Lu]Lu-EDTMP for pain palliation in patients with bone metastases secondary to breast and prostate carcinoma have delivered encouraging results [16–21]. Radiation dosimetry analysis has also shown its safety with low dose delivery to the kidneys in patients with breast carcinoma and mCRPC in comparison with other bone pain palliating agents in use [5, 7, 26]. However, the lower kinetic stability of [^177^Lu]Lu-EDTMP requires a high ligand concentration which is a drawback [2]. Also, [^68^Ga]Ga-EDTMP showed lower skeletal accumulation compared with its [^177^Lu]Lu-EDTMP analogue and could not be paired as a theranostic agent. In contrast, DOTA-conjugated theranostic bisphosphonates, such as [^177^Lu]Lu-BPAMD and [^68^Ga]Ga-BPAMD, have shown excellent results and represent good theranostic pairs [23].

Zoledronate, nitrogen containing hydroxy bisphosphonate, has recently been conjugated with DOTA (DOTA^ZOL^) and labeled with gallium-68 for imaging and with lutetium-177 for therapy as new theranostic pair for targeting bone metastases [27]. Zoledronate is known to have improved antiresorptive effects owing to its higher hydroxyapatite binding and internalization by osteoclasts with subsequent increased apoptosis. The mode of action is accompanied by inhibition of farnesyl pyrophosphate enzyme of the mevalonate pathway that results in the inhibition of osteoclastic activity [13]. Data from animal studies comparing the biodistribution and dosimetric analysis extrapolated for humans between [^177^Lu]Lu-EDTMP and [^177^Lu]Lu-DOTA^ZOL^ indicate a higher skeletal absorbed dose of 12.7 ± 1.018 for trabecular bone surface and 9.524 ± 0.803 for cortical bone surface with [^177^Lu]Lu-DOTA^ZOL^ compared with 10.019 ± 0.714 for cortical bone surface and 7.839 ± 0.655 for trabecular bone surface with [^177^Lu]Lu-EDTMP, hence presenting [^177^Lu]Lu-DOTA^ZOL^ to be a better agent for radionuclide therapy of bone metastases [28]. Recently, [^68^Ga]Ga-NODAGA^ZOL^ and [^177^Lu]Lu-DOTA^ZOL^ have been reported as the most effective new bisphosphonate based theranostic radiopharmaceuticals [2, 3, 27–30]. Our current study aims at first in human biodistribution and dosimetric analysis of [^177^Lu]Lu-DOTA^ZOL^ in mCRPC and metastasized bronchial carcinoma patients.

## Patients and methods

### Patient selection

In this retrospective study, we analyzed four patients with metastatic skeletal disease secondary to mCRPC or bronchial carcinoma that were treated between July 2016 and September 2017 with [^177^Lu]Lu-DOTA^ZOL^. All treatments were performed in the context of an individual treatment attempt as no other treatment options were left for these patients. Written informed consent was obtained from all patients. The local ethical committee waived the ethical statement due to the retrospective character of the study. All procedures were followed in accordance with ethical standards of the institutional review board and therefore been performed in accordance with the ethical standards laid down in the 1964 Declaration of Helsinki and all subsequent revisions.

**Fig. 1:**
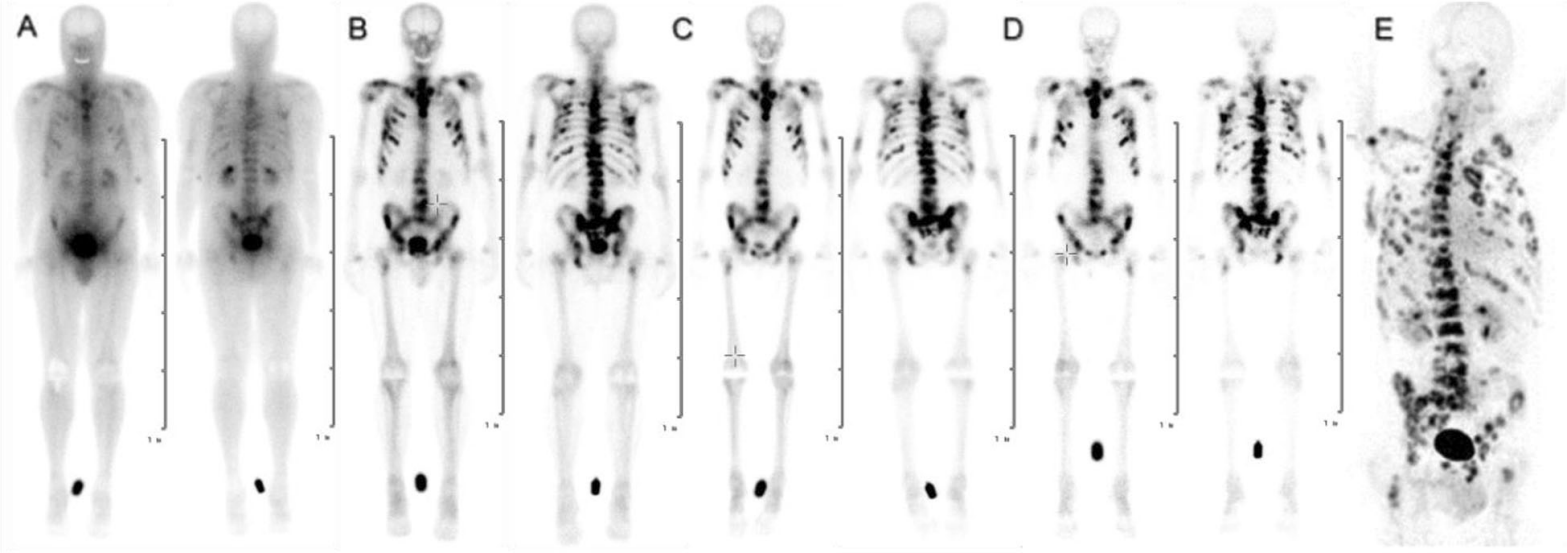
Planar scintigraphy (anterior and posterior views) after therapeutic application of [^177^Lu]Lu-DOTA^ZOL^ at **a** 20 min, **b** 3 h, **c** 24 h, **d** 168 h, and **e** PET/CT after application of [^68^Ga]Ga-DOTA^ZOL^ in a patient with bone metastases secondary to bronchial carcinoma (patient no. 3)

After confirming sufficient uptake in the bone metastases with [^68^Ga]Ga-DOTA^ZOL^ PET/CT shown in Fig. 1e, patients were hospitalized in our treatment unit. All patients had normal kidney function confirmed by renal function tests and renal scintigraphy. Patients were administered a mean activity of 5968 MBq (161.3 mCi) (5873–6000 MBq) of [^177^Lu]Lu-DOTA^ZOL^ intravenously. Table 1 shows patient details along with a history of previous treatments.

**Table 1:** Subject details of patients receiving radionuclide therapy with [^177^ Lu]Lu-DOTA^ZOL^

	PT1	PT2	PT3	PT4	Mean	SD
Age	83	66	64	64	69.25	9.22
BSA	1.922	1.976	1.979	1.979	1.964	0.028
Hemoglobin (g/dl)	14.3	9.6	13.1	12.6	12.4	2
Thrombocytes (G/l)	187	394	226	189	249	98.32
Leucocytes (G/l)	5.47	9.77	5.37	3.35	5.99	2.70
Hematocrit	0.4	0.4	0.4	0.37	0.39	0.02
Dose (MBq)	6000	6000	5873	6000	5968.25	63.50
Tumor	mCRPC	mCRPC	Bronchial carcinoma	Bronchial carcinoma		
Previous therapies received	Degarelix/denusumab [^177^77Lu]Lu-PSMA-617	Local Irradiation/docetaxel leuprorelin acetate, abiraterone/denosumab	Carboplatin/denosumab, nivolumab	Carboplatin/denosumab, nivolumab		
Extent of metastases	Extensive	Extensive	Extensive	Extensive		
ECOG status	0	0	0	0		

### Preparation of [^177^Lu]Lu-DOTA^ZOL^

**Fig. 2:**
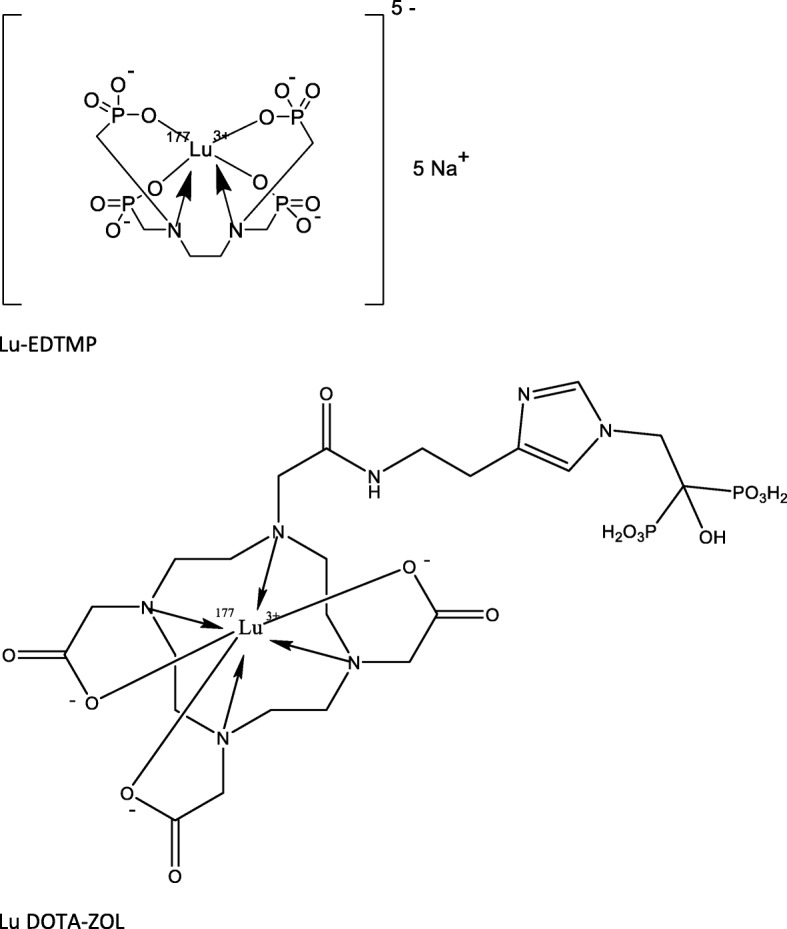
Comparison of chemical structures of [^177^Lu]Lu-DOTA^ZOL^ and [^177^Lu]Lu-EDTMP

DOTA^ZOL^ was radiolabeled in 0.8 ml ascorbic buffer (210 mg Na-L-ascorbat +42 mg gentisic acid in 1 ml 0.05 N HCl) with non-carrier-added lutetium-177, both obtained from ITG Isotope Technologies Garching GmbH, Garching, Germany. Manual synthesis was carried out on a thermoshaker at a temperature of 95 °C for 30 min. For quality control, an aliquot was retained from the final formulation. Quality control was performed with silica gel–coated aluminum TLC plates (silica 60 F254.5 × 4.5 cm, Merck, Darmstadt, Germany). Analysis was performed with a single trace radio-TLC scanner (PET-miniGITA, Elysia-Raytest, Straubenhardt, Germany) and evaluation software (GinaStar TLC, Elysia-Raytest, Straubenhardt, Germany). Development of silica TLC plates was conducted in 0.1 M citrate buffer (pH 4) mobile phase, whether [^177^Lu]Lu-DOTA^ZOL^ is found at R/F: 0–0.1 and DOTA at R/F: 0.5. Second, developed in 1 M acetylaceton/aceton mixture (1:1) as mobile phase, where [^177^Lu]Lu-DOTA-ZOL is found at 0–0.1 and unlabelled lutetium-177 at 0.8–1. At last, a phosphate buffer with MeOH 19:1 as a mobile phase, where lutetium-177 colloide is found at 0–0.2 and [^177^Lu]Lu-DOTA^ZOL^ at 0.8–1. Radio-HPLC was used to determine the radiochemical purity, especially the content of radiolysis products as well as unlabelled gallium-68. RadioHPLC was performed using Agilent 1260 Infinity II reverse phase HPLC system (Agilent Technologies, Santa Clara, California) equipped with GABI γ-HPLC flow detector (Elysia-raytest, Straubenhardt, Germany) and a PC interface running Gina Star software (Elysia-raytest, Straubenhardt, Germany). A Nucleodur 100-3 C18 ec 250/4 column (Macherey-Nagel GmbH & Co. KG, Düren, Germany) was used. The gradient elution system utilized mobile phase A (Puffer) and mobile phase B (MeOH) at a flow rate of 0.5 mL/min isocratic with 90% phase A and 10% phase B. Radioactivity was measured with a dose calibrator (ISOMED 2010, MED Nuklear-Medizintechnik Dresden GmbH, Dresden, Germany). A radiochemical yield of ≥ 95% and a radiochemical purity ≥ 98% was obtained. A comparison of the chemical structures of [^177^Lu]Lu-DOTA^ZOL^ and [^177^Lu]Lu-EDTMP can be found in Fig. 2.

### Imaging protocol

Serial whole body planar scintigraphy (anterior and posterior views) was performed with dual head Symbia SPECT/CT system (Symbia T, Siemens Healthineers, Erlangen, Germany) at 20 min and 3, 24, and 167 h post injection (p.i). Acquisition was done in the supine position at a speed of 10 cm/min using medium energy parallel hole collimators with 20% energy window centered at a photopeak of 208 keV. Images were processed using an iterative ordered subset maximization algorithm provided by the manufacturer into a matrix of 256 × 1024. The first data set obtained at 20 min (prior to voiding of the bladder) was considered as a reference with 100% of administered activity. A standard source of known activity was placed between the legs in all images at the time of acquisition. For conversion of counts/min to activity, the gamma camera was pre-calibrated using a known activity of [^177^Lu]Lu-DOTA^ZOL^ and imaging it at the same speed and distance of 10 cm/min.

### Blood sampling

One to two milliliters of blood samples was drawn at 20 min and 3, 8, 24, and 167 h p.i. Due to high counts that may lead to errors in measurement, 0.2-ml blood samples were prepared and measured along with 0.2-ml sample from the known standard activity of [^177^Lu]Lu-DOTA^ZOL^ using a 1480 WIZARD^TM^ 3n Gamma counter. The calibration factor determined from the standard activity measurement was used to determine the activity in blood samples at the respective data points.

### Data analysis

The images were qualitatively analyzed to assess the biodistribution of [^177^Lu]Lu-DOTA^ZOL^ in the whole body and organs. All organs with uptake equal to or more than that of the kidneys were considered as source organs. Kidneys and urinary bladder showed high uptake in the 24-h image data sets and were considered as source organs that included kidneys and urinary bladder. Adductor muscle was measured as soft tissue reference. Whole body ROI’s were drawn. A rectangular ROI was drawn near the head region above the shoulder for background measurement and an elliptical ROI was used for measurement of the standard source placed between the legs. Same sized ROI’s were replicated on serial images (kidneys ROi’s up to the 24-h data set and all remaining ROI’s in all subsequent image data sets).

Background corrected counts in the right and left kidney, soft tissue, urinary bladder, and whole body were determined on anterior and posterior images. The geometric mean counts/min in all source organs at all data time points was determined. Using EANM dosimetry committee guidelines [31], whole body activity at subsequent time points (T) was determined by multiplying the injected activity with the normalized geometric mean whole body counts at the respective time points as given in Eq. 1.

1 $$ {A}_{WB,T}={A}_0\frac{\sqrt{{\mathrm{Anterior}\ \mathrm{counts}}_{\mathrm{T}}.\mathrm{Posterior}\ {\mathrm{counts}}_{\mathrm{T}}}}{\sqrt{{\mathrm{Anterior}\ \mathrm{counts}}_{\mathrm{t}}.{\mathrm{Posterior}\ \mathrm{counts}}_{\mathrm{t}}}} $$

where *t* = 20 min, *T* = subsequent time points, and *A*_0_ = initial injected activity. Likewise, activity in the urinary bladder was also determined by multiplying the injected activity with the normalized geometric mean counts in the urinary bladder.

For calculation of activity in the right and left kidneys at all data points, a conjugate view method with a simple geometrically based subtraction technique given in Eq. 2 [32] was used.

2 $$ {A}_j=\sqrt{\frac{I_A{I}_P}{e^{-{\mu}_et}}}\frac{f_j}{C} $$

where *I*_*A*_ = anterior count rate, *I*_*P*_ = posterior count rate, *f*_*j*_ represents source organ self-attenuation correction which was calculated from the source region linear attenuation coefficient *μ*_*j*_, and source thickness *t*_*j*_ using Eq. 3 [32]. Factor *μ*_*e*_*t* represents the transmission factor across the patient thickness *t* in the area of the ROI with a linear attenuation coefficient *μ*_*e*_ calculated using Eq. 4 [32]. From [^68^Ga]Ga-DOTA^ZOL^- PET/CT of a respective patient, CT-based measurements of source organ thickness as well as whole body thickness and thickness anterior and posterior to source organs at the same level were used. *C* is the calibration factor determined for gamma camera with a known standard source and was the same in all the studies. For the measurements of *μ*_*j*_ and *μ*_*i*_ (linear attenuation coefficients for whole thickness), we applied a CT-based Hounsfield unit method described by Kabasakal et al. [33] for [^177^Lu]Lu-PSMA-617 dosimetric analysis.

3 $$ {f}_j=\frac{\left(\frac{\mu_j{t}_j}{2}\right)}{\sinh \left(\frac{\mu_j{t}_j}{2}\right)} $$

4 $$ {\mu}_e=\left(\frac{1}{t}\right)\sum \limits_{i=1}^n{\mu}_i{t}_i={\mu}_j+\left(\frac{1}{t}\right)\sum \limits_{i=1}^n\left({\mu}_i-{\mu}_j\right){t}_i $$

A simple geometric based background subtraction technique using Eq. 5 [32] was used.

5 $$ F={\left\{\left[1-\left(\frac{I_{ADJ}}{I_A}\right)\left(1-\frac{t_j}{t}\right)\right]\left[1-\left(\frac{I_{ADJ}}{I_P}\right)\left(1-\frac{t_j}{t}\right)\right]\right\}}^{\frac{1}{2}} $$

where *I*_*ADJ*_ is the count rate through the patient from a soft tissue area of the same size as that of the organ ROI. *I*_*A*_*, I*_*P*_*, t*_*j,*_ and *t* are the same as previously defined.

Percent injected activity in the whole body, urinary bladder, and kidneys at all time points was determined. To calculate percent injected activity in the skeleton, from percent whole body activity, percent blood, urinary bladder, and kidneys activities were subtracted.

### Dosimetric analysis

Percent injected activity in the whole body, kidneys, and skeletal system at all data time points was used to determine residence times (MBq-h/MBq) by fitting biexponential kinetic analysis using OLINDA/EXM version 2.0 (Hermes Medical Solutions, Stockholm, Sweden) software in these organs. The residence times for the skeletal system were assumed to be distributed equally between trabecular and cortical bone. An indirect blood-based method using patient-based red marrow-to-blood ratio (RMBLR) and bone marrow mass was used to determine bone marrow self-dose [34, 35].

Urinary excretion fraction at all time points was determined by applying the function A_0_(1 − e^−ƛ,T^). With a logarithmic function fit on the urinary excretion curve, effective excretion half-life was obtained. Using the total urinary excretion fraction, effective excretion half-life and 4-h voiding interval as input in Cloutier’s dynamic urinary bladder model, residence time for urinary bladder contents was obtained. By subtracting residence times for kidneys, bone marrow, and skeletal system from whole body residence time, the remainder of body residence time was calculated.

Residence time for kidneys, cortical and trabecular bone, urinary bladder contents, red marrow self-dose, and remainder of body were used as an input in OLINDA/EXM version 2.0 (Hermes Medical Solutions, Stockholm, Sweden) software for calculation of organ absorbed doses and effective doses after adjusting the weight of patient organs by multiplying the reference adult male weight with factor obtained by dividing patient weight with the reference adult male weight. The mean of residence times and organ absorbed doses (mSv/MBq) were calculated.

## Results

### Qualitative analysis

**Fig. 3:**
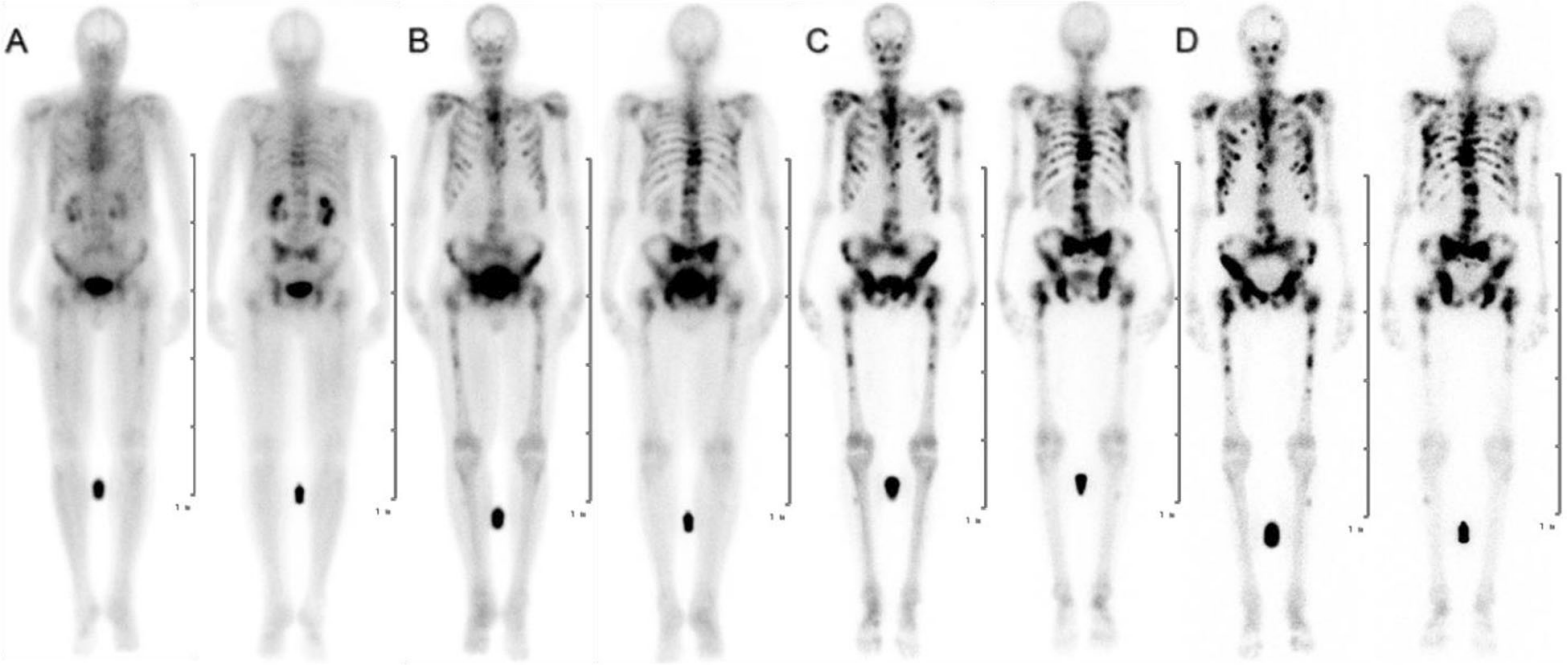
Planar scintigraphy (anterior and posterior views) after therapeutic application of [^177^Lu]Lu-DOTA^ZOL^ at **a** 20 min, **b** 3 h, **c** 24 h, and **d** 168 h in a patient with bone metastases secondary to prostate cancer (patient no. 1)

Figures 1 and 3 show the biodistribution of [^177^Lu]Lu-DOTA^ZOL^ in one patient with bronchial carcinoma and one patient with mCRPC, respectively. In the initial 20 min p.i. image data set, highest uptake was seen in the urinary bladder followed by kidneys and soft tissue with minimal uptake in the skeletal system. The kidneys showed a rapid decrease in activity at 3 h with minimum to no uptake after 24 h p.i. The intense uptake was seen in the skeletal system from 3 h onwards. Blood and soft tissue clearance and lesion to normal bone contrast increased in later images up to 168 h. The mean ± SD 24-h whole body retention was found to be 31.25 ± 6.5.

In this small patient study, we observed fast uptake and clearance kinetics of kidneys in patients with bronchial carcinoma compared with mCRPC patient, which resulted in better skeletal to soft tissue contrast as early as 3 h p.i. in the bronchial carcinoma patient compared with 24 h p.i. in mCRPC patient.

### Quantitative analysis

Mean residence times (MBq-h/MBq) (Table 2) was found to be highest in trabecular and cortical bone (31.9 h) followed by the remainder of the body (11.7 h), kidneys (1.84 h), urinary bladder (1.52 h), and bone marrow (0.03 h). In patient no. 1, residence times for the skeletal system and the kidney were lower compared with the other patients.

**Table 2:** Residence times (MBq-h/MBq) of [^177^ Lu]Lu-DOTA^ZOL^ in comparison with [^177^Lu]Lu-EDTMP [5]

Organs	[^177^Lu]Lu-DOTA^ZOL^						[^177^Lu]Lu-EDTMP [5]
	PT1	PT2	PT3	PT4	Mean	± SD	
Kidneys	0.96	2.01	2.43	1.96	1.84	0.63	
Trabecular bone	27.45	34.95	33.45	31.85	31.93	3.24	48.62
Cortical bone	27.45	34.95	33.45	31.85	31.93	3.24	48.62
Red marrow	0.01	0.03	0.04	0.03	0.03	0.01	0.07
Urinary bladder contents	1.59	1.48	1.51	1.50	1.52	0.05	7.23
Remainder of body	0.23	44.06	0.93	1.40	11.65	21.61	27.37
Whole body	56.10	116.00	70.30	67.10	77.38	26.46	131.91
Blood	0.19	0.34	0.35	0.46	0.34	0.11	0.98

Mean organ absorbed doses (Table 3) were found highest (3.33 ± 0.35 mSv/MBq) for osteogenic cells, followed by kidneys (0.49 ± 0.16 mSv/MBq), red marrow (0.461 ± 0.064 mSv/MBq), and urinary bladder wall (0.322 ± 0.022 mSv/MBq). Kidney and osteogenic cell absorbed doses were lowest in patient no. 1. The mean total body dose was 0.092 ± 0.033 mSv/MBq.

**Table 3:** Organ absorbed doses (mSv/MBq) of [^177^Lu]Lu-DOTA^ZOL^

Organs	PT1	PT2	PT3	PT4	Mean	± SD
Adrenals	0.010	0.069	0.016	0.015	0.027	0.028
Brain	0.007	0.061	0.009	0.009	0.021	0.027
Esophagus	0.004	0.060	0.006	0.006	0.019	0.027
Eyes	0.007	0.061	0.009	0.009	0.021	0.027
Gall bladder wall	0.003	0.060	0.005	0.005	0.018	0.028
Left colon	0.005	0.062	0.007	0.007	0.020	0.028
Small intestine	0.004	0.061	0.006	0.006	0.019	0.028
Stomach wall	0.003	0.058	0.004	0.004	0.017	0.027
Right colon	0.003	0.060	0.005	0.005	0.018	0.028
Rectum	0.006	0.062	0.007	0.007	0.021	0.028
Heart wall	0.003	0.059	0.005	0.005	0.018	0.027
Kidneys	0.263	0.555	0.632	0.511	0.490	0.160
Liver	0.003	0.059	0.005	0.005	0.018	0.027
Lungs	0.004	0.059	0.005	0.006	0.019	0.027
Pancreas	0.004	0.061	0.006	0.006	0.019	0.028
Prostate	0.005	0.060	0.006	0.006	0.019	0.027
Salivary glands	0.004	0.060	0.006	0.006	0.019	0.027
Red marrow	0.402	0.551	0.456	0.434	0.461	0.064
Osteogenic cells	2.940	3.770	3.330	3.170	3.300	0.350
Spleen	0.004	0.060	0.006	0.006	0.019	0.027
Testes	0.003	0.057	0.004	0.004	0.017	0.027
Thymus	0.003	0.058	0.004	0.005	0.017	0.027
Thyroid	0.004	0.060	0.006	0.006	0.019	0.027
Urinary bladder wall	0.333	0.364	0.316	0.316	0.332	0.023
Total body	0.069	0.142	0.081	0.077	0.092	0.034

## Discussion

Zoledronate presents as an ideal candidate for labeling with the therapeutic radionuclide lutetium-177 for radionuclide therapy of bone metastases, as it shows high osteoclast and hydroxyl apatite binding [27] and no in vivo biotransformation [3]. Preclinical small animal studies using [^177^Lu]Lu-DOTA^ZOL^ and [^68^Ga]Ga-DOTA^ZOL^ showed comparable results, suggesting the two tracers as new theranostic pair for bone-targeted radionuclide therapy [27].

Extrapolation of dosimetric analysis of [^177^Lu]Lu-DOTA^ZOL^ and [^177^Lu]Lu-EDTMP from rats to humans revealed high kidney and trabecular bone absorbed doses as well as high trabecular bone to other organs absorbed dose ratios for [^177^Lu]Lu-DOTA^ZOL^ [28]. The higher thermodynamic and kinetic stability, leading to high bone uptake with low soft tissue accumulation, suggests [^177^Lu]Lu-DOTA^ZOL^ to be a better therapeutic bisphosphonate compared with [^177^Lu]Lu-EDTMP [2].

**Fig. 4:**
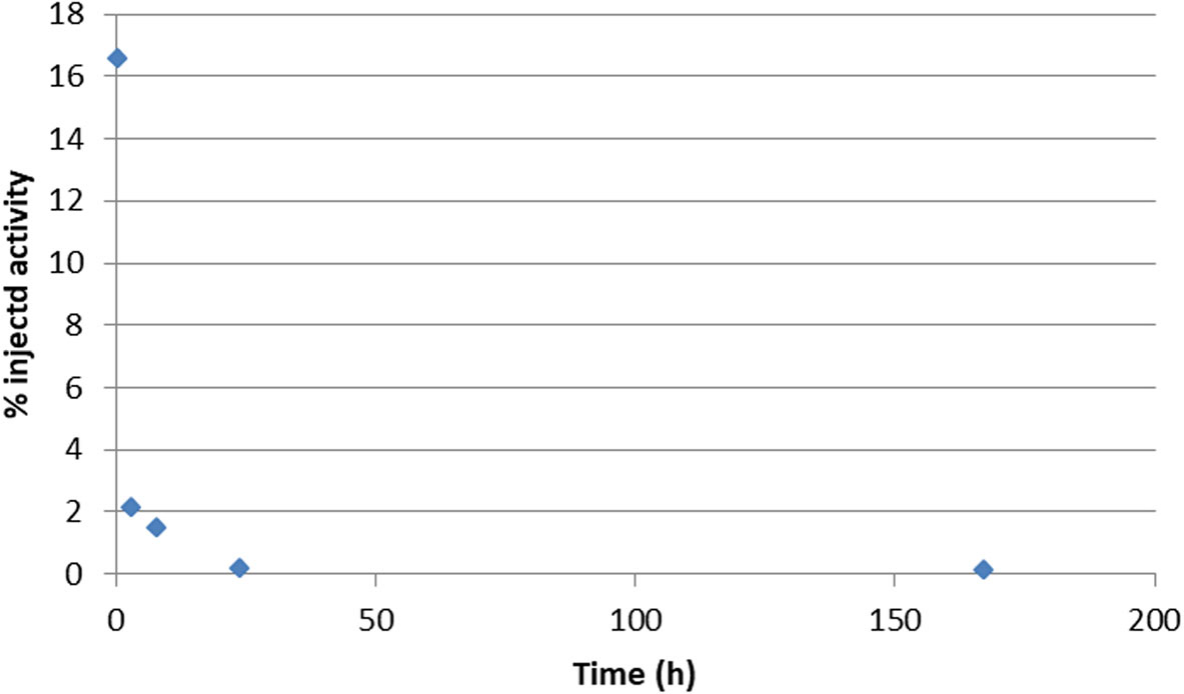
Blood clearance curve in patient no. 4 with bronchial carcinoma

The current study is the first ever human biodistribution and dosimetric analysis for [^177^Lu]Lu-DOTA^ZOL^ in mCRPC and bronchial carcinoma patients. We noticed that the biodistribution of [^177^Lu]Lu-DOTA^ZOL^ in humans (Figs. 1 and 3) is consistent with preclinical biodistribution studies in male Wistar rats [27, 28]. We found the highest accumulation in the skeleton with fast kidney uptake and clearance. As the kidneys are the sole route of its excretion, the urinary bladder showed high uptake as well. Blood and soft tissue showed rapid clearance which resulted in good skeleton to soft tissue contrast. A rapid and biphasic blood clearance curve was found (Fig. 4) comparable to [^177^Lu]Lu-EDTMP [5]. No uptake was seen in any other organ.

Prominent uptake in the skeletal system in bronchial carcinoma patients was visualized at 3 h p.i. image in contrast to 24 h p.i. in mCRPC patients. The finding of best bone-to-soft tissue contrast at 24 h p.i. in mCRPC patients is consistent with similar observations with [^177^Lu]Lu-EDTMP distribution in mCRPC patients [5, 7, 26]. To establish whether the early uptake in bronchial carcinoma patients is a patient dependent or tumor dependent finding and can be of any significance in relation to tumor lesion doses needs further large scale and tumor lesion dosimetry studies.

The source organs identified for dosimetric analysis included the kidneys, bone marrow, urinary bladder, skeletal system, and the whole body. A biphasic kinetic behavior of [^177^Lu]Lu-DOTA^ZOL^ was observed in all source organs and the whole body. Hence, biexponential curve fitting was used for residence time calculations. Residence time was highest in the skeleton similar to [^177^Lu]Lu-EDTMP (Table 2) [5]. Residence time for all source organs except the kidneys were lower in comparison with [^177^Lu]Lu-EDTMP (Table 2) [5]. The low number of patients and the different methodologies used for the determination of residence time in our current study might be causes for this difference. However, the ratio of skeletal to whole body residence time was higher for [^177^Lu]Lu-DOTA^ZOL^ compared with [^177^Lu]Lu-EDTMP [5].

**Fig. 5:**
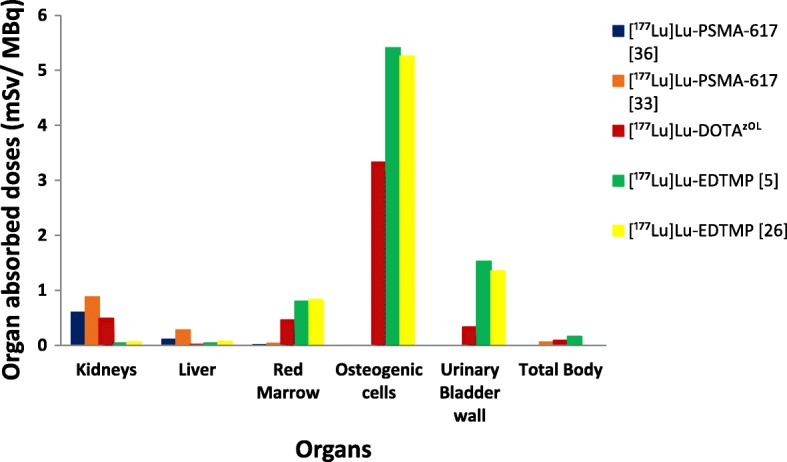
Comparison of organ absorbed doses of [^177^Lu]Lu-DOTA^ZOL^ with [^177^Lu]Lu-EDTMP [5, 26], [^177^Lu]Lu-PSMA-617 [33, 36].

We found lower mean organ absorbed doses for osteogenic cells (3.33 ± 0.35 mSv/MBq) compared with 5.41 and 5.26 mSv/MBq reported for [^177^Lu]Lu-EDTMP [5, 26], (Fig. 5) as well as 4.04 mSv/MBq for [^153^Sm]Sm-EDTMP [26]. The difference might be due to humerus [5] or femoral [26] activity extrapolation for skeletal activity and residence time calculations for [^177^Lu]Lu-EDTMP compared with the calculation of skeletal activity by deduction of percent kidney, blood, and bladder activity from percent whole body activity in the current study.

In our study, we found a higher mean organ absorbed dose for the kidneys (0.49 mSv/MBq) compared with [^177^Lu]Lu-EDTMP (0.04 and 0.06 mSv/MBq) [5, 26]. In contrast to the use of the conjugate view method for kidney residence time calculation in our current study, Bal et al. [5] neglected kidney self-dose in absorbed dose determination and Sharma et al. [26] used a different methodology for calculation of percent injected doses in kidneys which resulted in lower kidney dose for [^177^Lu]Lu-EDTMP. Hence, the kidney absorbed doses reported for [^177^Lu]Lu-EDTMP cannot be compared with the results of [^177^Lu]Lu-DOTA^ZOL^ in our current study. The mean organ absorbed dose to the urinary bladder wall (0.332 mSv/MBq) was found to be lower in our study compared with [^177^Lu]Lu-EDTMP (1.53 mSv/MBq) [5, 26]. This difference could be due to the use of Cloutier’s method with cumulative urinary calculation from whole body retention and 4-h voiding intervals in our current study compared with the collection of urine samples for residence time calculation in the [^177^Lu]Lu-EDTMP studies.

[^177^Lu]Lu-DOTA^ZOL^ resulted in a lower bone marrow absorbed dose compared with [^177^Lu]Lu-EDTMP [5, 7, 26], which in theory allows administration of higher therapeutic activities of [^177^Lu]Lu-DOTA^ZOL^. Based on a maximum permissible radiation absorbed dose to the bone marrow of 2Gy, the maximum tolerated dose for [^177^Lu]Lu-DOTA^ZOL^ is estimated to be 3630-4980 MBq compared with 2000–3250 MBq for [^177^Lu]Lu-EDTMP [5]. As a result, radiation absorbed dose of 11 to 16 Gy will be delivered to osteogenic cells by [^177^Lu]Lu-DOTA^ZOL^ which is comparable with 10.1 to 17.6 Gy for [^177^Lu]Lu-EDTMP. Using these thresholds, the kidney absorbed dose remains well below the maximum permissible dose limit of 23 Gy.

As absorbed dose to kidneys is one of the important factors in radionuclide therapy using Lutetium-177-labeled radiopharmaceuticals, we found that [^177^Lu]Lu-DOTA^ZOL^ delivers a lower (by a factor of 1.2 to 1.88) kidney dose (Fig. 5) in comparison with [^177^Lu]Lu-PSMA-617 [33, 36].

Further, we would like to add here that in this study we used complementary PET/CT images of the patient for organ and whole body thickness calculation. However, whole body SPECT/CT may result in similar images. Though time consuming and inconvenient for the seriously ill patients, these images can still be of added value in future dosimetry studies.

## Conclusion

[^177^Lu]Lu-DOTA^ZOL^ is a promising new therapeutic radiopharmaceutical for radionuclide therapy of bone metastases due to excellent skeletal uptake, a lower low bone marrow dose than [^177^Lu]Lu-EDTMP and a very low kidney dose. Labeling with gallium-68 delivers [^68^Ga]Ga-DOTA^ZOL^, which represents an ideal theranostic counterpart for PET/CT imaging. Further studies are warranted to evaluate the efficacy and safety of radionuclide therapy with [^177^Lu]Lu-DOTA^ZOL^ in the clinical setting.

## Data Availability

The datasets used and/or analyzed during the current study are available from the corresponding author on reasonable request.

## References

[1] Pandit-Taskar N, Batraki M, Divgi CR (2004). Radiopharmaceutical therapy for palliation of bone pain from osseous metastases. J Nucl Med..

[2] Bergmann R, Meckel M, Kubíček V, Pietzsch J, Steinbach J, Hermann P (2016). ^177^Lu-labelled macrocyclic bisphosphonates for targeting bone metastasis in cancer treatment. EJNMMI Res..

[3] Nikzad M, Jalilian AR, Shirvani-Arani S, Bahrami-Samani A, Golchoubian H (2013). Production, quality control and pharmacokinetic studies of ^177^Lu-zoledronate for bone pain palliation therapy. J Radioanal Nucl Chem..

[4] Ogawa K, Ishizaki A (2015). Well-designed bone-seeking radiolabeled compounds for diagnosis and therapy of bone metastases. Biomed Res Int..

[5] Bal C, Arora G, Kumar P, Damle N, Das T, Chakraborty S (2016). Pharmacokinetic, dosimetry and toxicity study of ^177^Lu-EDTMP in patients: phase 0/I study. Curr Radiopharm..

[6] Fakhari A, Reza Jalilian A, Yousefnia H, Bahrami-Samani A, Johari-Daha F, Khalaj A (2015). Radiolabeling and evaluation of two 177 Lu-labeled bis-phosphonates preparation of two ^177^Lu-labeled bis-phosphonates. Iran J Nucl Med..

[7] Balter H, Victoria T, Mariella T, Javier G, Rodolfo F, Andrea P (2015). ^177^ Lu-labeled agents for neuroendocrine tumor therapy and bone pain palliation in Uruguay. Curr Radiopharm.

[8] Kam BLR, Teunissen JJM, Krenning EP, De Herder WW, Khan S, Van Vliet EI (2012). Lutetium-labelled peptides for therapy of neuroendocrine tumours. Eur J Nucl Med Mol Imaging..

[9] Frilling A, Weber F, Saner F, Bockisch A, Hofmann M, Mueller-Brand J (2006). Treatment with ^90^Y- and ^177^Lu-DOTATOC in patients with metastatic neuroendocrine tumors. Surgery..

[10] Ahmadzadehfar H, Eppard E, Kürpig S, Fimmers R, Yordanova A, Schlenkhoff CD (2016). Therapeutic response and side effects of repeated radioligand therapy with ^177^Lu-PSMA-DKFZ-617 of castrate-resistant metastatic prostate cancer. Oncotarget..

[11] Pfestroff A, Luster M, Jilg CA, Olbert PJ, Ohlmann CH, Lassmann M (2015). Current status and future perspectives of PSMA-targeted therapy in Europe: opportunity knocks. Eur J Nucl Med Mol Imaging..

[12] Yordanova A, Eppard E, Kürpig S, Bundschuh RA, Schönberger S, Gonzalez-Carmona M (2017). Theranostics in nuclear medicine practice. OncoTargets Ther..

[13] Russell RGG (2007). Bisphosphonates: mode of action and pharmacology. Pediatrics..

[14] Chakraborty S, Das T, Sarma HD, Venkatesh M, Banerjee S (2008). Comparative studies of ^177^Lu-EDTMP and 177Lu-DOTMP as potential agents for palliative radiotherapy of bone metastasis. Appl Radiat Isot..

[15] Das T, Shinto A, Kamaleshwaran KK, Banerjee S (2016). Theranostic treatment of metastatic bone pain with^177^Lu-DOTMP. Clin Nucl Med..

[16] Mazzarri S, Guidoccio F, Mariani G (2015). The emerging potential of ^177^Lu-EDTMP: an attractive novel option for radiometabolic therapy of skeletal metastases. Clin Transl Imaging..

[17] Shinto AS, Shibu D, Kamaleshwaran KK, Das T, Chakraborty S, Banerjee S (2014). ^177^Lu-EDTMP for treatment of bone pain in patients with disseminated skeletal metastases. J Nucl Med Technol..

[18] Agarwal KK, Singla S, Arora G, Bal C (2015). ^177^Lu-EDTMP for palliation of pain from bone metastases in patients with prostate and breast cancer: a phase II study. Eur J Nucl Med Mol Imaging..

[19] Alavi M, Omidvari S, Jalilian A, Mehdizadeh A, Bahrami-Samani A (2015). Metastatic bone pain palliation using ^177^ Lu-ethylenediaminetetramethylene phosphonic acid. World J Nucl Med..

[20] Thapa P, Nikam D, Das T, Sonawane G, Agarwal JP, Basu S (2015). Clinical efficacy and safety comparison of ^177^Lu-EDTMP with ^153^Sm-EDTMP on an equidose basis in patients with painful skeletal metastases. J Nucl Med..

[21] Yuan J, Liu C, Liu X, Wang Y, Kuai D, Zhang G (2013). Efficacy and safety of ^177^Lu-EDTMP in bone metastatic pain palliation in breast cancer and hormone refractory prostate cancer. Clin Nucl Med..

[22] Baum RP, Kulkarni HR (2012). THERANOSTICS: from molecular imaging using ga-68 labeled tracers and pet/ct to personalized radionuclide therapy - the Bad Berka experience. Theranostics..

[23] Pfannkuchen N, Meckel M, Bergmann R, Bachmann M, Bal C, Sathekge M (2017). Novel radiolabeled bisphosphonates for PET diagnosis and endoradiotherapy of bone metastases. Pharmaceuticals..

[24] Rösch F, Baum RP (2011). Generator-based PET radiopharmaceuticals for molecular imaging of tumours: on the way to THERANOSTICS. Dalton Trans..

[25] Passah A, Tripathi M, Ballal S, Yadav MP, Kumar R, Roesch F (2017). Evaluation of bone-seeking novel radiotracer ^68^Ga-NO2AP-bisphosphonate for the detection of skeletal metastases in carcinoma breast. Eur J Nucl Med Mol Imaging..

[26] Sharma S, Singh B, Koul A, Mittal BR (2017). Comparative therapeutic efficacy of ^153^Sm-EDTMP and ^177^Lu-EDTMP for bone pain palliation in patients with skeletal metastases: patients’ pain score analysis and personalized dosimetry. Front Med.

[27] Meckel M, Bergmann R, Miederer M, Roesch F (2017). Bone targeting compounds for radiotherapy and imaging: *Me(III)-DOTA conjugates of bisphosphonic acid, pamidronic acid and zoledronic acid. EJNMMI Radiopharm Chem..

[28] Yousefnia H, Zolghadri S, Jalilian A (2015). Absorbed dose assessment of ^177^Lu-zoledronate and ^177^Lu-EDTMP for human based on biodistribution data in rats. J Med Phys..

[29] Pfannkuchen N, Bausbacher N, Pektor S, Miederer M, Rosch F (2018). In vivo evaluation of [ ^225^ Ac]Ac-DOTA ZOL for α-therapy of bone metastases. Curr Radiopharm..

[30] Holub J, Meckel M, Kubíček V, Rösch F, Hermann P (2015). Gallium(III) complexes of NOTA-bis (phosphonate) conjugates as PET radiotracers for bone imaging. Contrast Media Mol Imaging..

[31] Hindorf C, Glatting G, Chiesa C, Lindén O, Flux G (2010). EANM dosimetry committee guidelines for bone marrow and whole-body dosimetry. Eur J Nucl Med Mol Imaging..

[32] Siegel J, Thomas SR (1999). MIRD pamphlet no. 16: Techniques for quantitative radiopharmaceutical biodistribution data acquisition and analysis for use in human radiation dose estimates. J Nucl Med..

[33] Kabasakal L, Abuqbeitah M, Aygün A, Yeyin N, Ocak M, Demirci E (2015). Pre-therapeutic dosimetry of normal organs and tissues of ^177^Lu-PSMA-617 prostate-specific membrane antigen (PSMA) inhibitor in patients with castration-resistant prostate cancer. Eur J Nucl Med Mol Imaging..

[34] Siegel JA (2005). Establishing a clinically meaningful predictive model of hematologic toxicity in nonmyeloablative targeted radiotherapy: practical aspects and limitations of red marrow dosimetry. CANCER Biother Radiopharm..

[35] Hindorf C, Lindén O, Tennvall J, Wingårdh K, Strand SE (2005). Evaluation of methods for red marrow dosimetry based on patients undergoing radioimmunotherapy. Acta Oncol (Madr).

[36] Scarpa L, Buxbaum S, Kendler D, Fink K, Bektic J, Gruber L (2017). The ^68^Ga/^177^Lu theragnostic concept in PSMA targeting of castration-resistant prostate cancer: correlation of SUVmax values and absorbed dose estimates. Eur J Nucl Med Mol Imaging..

